# Chemotherapy-induced nausea and vomiting in the literature: a cross-sectional analysis by time and emetogenicity

**DOI:** 10.1007/s00520-026-10934-1

**Published:** 2026-06-30

**Authors:** Ronald Chow, Daniel Zhang, Suhangi Brahmbhatt, Jennifer Leigh, Florian Scotté

**Affiliations:** 1 Temerty Faculty of Medicine, University of Toronto, Toronto, Canada; 2 Centre for Evidence-Based Medicine, University of Oxford, Oxford, UK; 3 Siebel School of Computing and Data Science, University of Illinois Urbana-Champaign, Champaign, IL USA; 4 Département Interdisciplinaire D’Organisation Des Parcours Patients, Gustave-Roussy, Université Paris-Saclay, 94800 Villejuif, France

**Keywords:** Chemotherapy-induced nausea and vomiting, Chemotherapy emetogenicity, Antiemetic therapy

## Abstract

Chemotherapy-induced nausea and vomiting (CINV) has been studied for decades, yet the overall scope and evolution of this literature have not been quantified. We conducted a cross-sectional survey of primary research articles on CINV published from database inception through January 2026, categorizing studies by chemotherapy emetogenicity. Nearly 3000 studies were identified, with publication output peaking in the 2010s and focusing predominantly on highly emetogenic regimens. These trends reflect historical clinical priorities and advances in antiemetic therapy.

## Introduction

Chemotherapy-induced nausea and vomiting (CINV) is a common side effect, causing significant impact on quality of life and even leading to treatment non-adherence [1]. In the literature, CINV research describes incidence according to time of onset. Acute CINV is described as occurring within 0–24 h of treatment initiation, delayed as 24–120 h after initiation, and long-delayed as occurring beyond 120 h after initiation [2].

Substantial research has been conducted into CINV, describing its phenomena, characterizing chemotherapy agents according to emetic potential, and prescribing prophylactic and rescue regimens to treat CINV [3, 4]. However, the overall state of the literature has never been quantified. Studying the scope and trajectory of this literature can provide important historical appreciation of the literature and possibly lead to insights into future trajectory in CINV and other antineoplastic-induced nausea and vomiting (i.e., antibody drug conjugate-induced nausea and vomiting, immunotherapy-induced nausea and vomiting). The aim of this study is to describe the CINV literature and its trajectory over time.

## Methods

We searched Ovid Medline and Embase from inception to January 8th, 2026,using the keyword search term “chemotherapy-induced nausea vomiting”. All retrieved results underwent title and abstract screening in duplicate: once by a large language model (ChatGPT-5; DZ) and once by an independent human reviewer (RC). Abstracts that reported on primary research articles on CINV were classified based on emetogenicity class as per the 2023 MASCC/ESMO consensus recommendation [4]; studies reporting on drugs with multiple emetogenicities were rated according to their highest class. Data were reported descriptively.

## Results

**Fig. 1:**
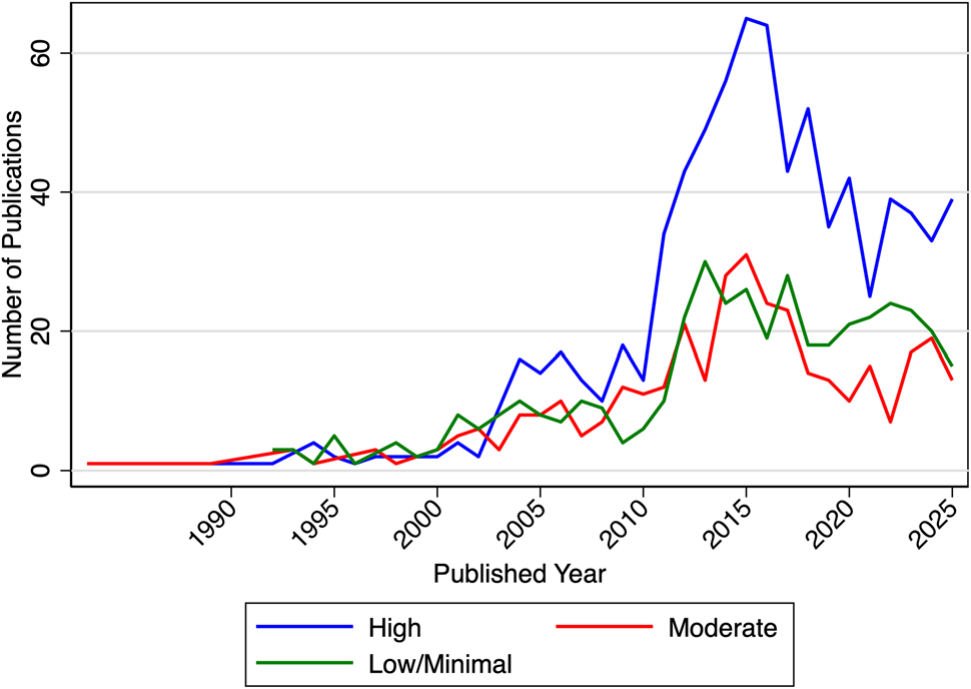
Distribution of studies over time, by emetogenicity

A total of 2922 primary research articles reporting on CINV were identified. Figure 1 illustrates the temporal trend in publication volume. The number of studies has increased progressively over time, most notably in the 2010s. Since 2020, there has been a decrease in the number of studies annually.

Highly emetogenic chemotherapies have been most studied, with 51% of studies; 22% reported on moderately emetogenic and 27% on low/minimally emetogenic chemotherapies. Fifty-six percent of studies were human clinical trials.

## Discussion

To our knowledge, this study is the first to comprehensively quantify the scope and temporal trajectory of the CINV literature, examining both publication trends over time and the distribution of studies by emetogenicity class. Nearly 3000 primary research articles were identified, with a steady rise in publication volume over several decades and a pronounced peak during the 2010s. Across all time periods, the literature has been dominated by studies of highly emetogenic chemotherapy regimens.

This pattern likely reflects enduring clinical priorities. Highly emetogenic chemotherapies pose the greatest risk of severe nausea and vomiting and have historically been associated with substantial morbidity, impaired quality of life, and treatment non-adherence. As a result, these regimens have naturally attracted the greatest research attention, particularly in the context of clinical trials aimed at establishing effective prophylactic and rescue antiemetic strategies. The comparatively smaller body of literature addressing moderately and low/minimally emetogenic regimens may reflect both lower symptom burden and fewer perceived unmet clinical needs in these populations.

The observed concentration of research on highly emetogenic chemotherapy may also reflect a bidirectional relationship between clinical guidelines and research activity. As guideline recommendations increasingly emphasized aggressive prophylaxis for high-risk regimens, these settings became preferential platforms for clinical trials and comparative studies. Conversely, lower-risk regimens—despite being more common in routine practice—may have been deprioritized, potentially limiting the evidence base for nuanced antiemetic optimization in these populations. This imbalance highlights an opportunity for future research to better align study designs with real-world chemotherapy utilization patterns.

The marked increase in publications during the 2000 s and 2010 s coincides with major advances in antiemetic therapy. The introduction and widespread adoption of neurokinin-1 (NK1) receptor antagonists, followed by growing evidence supporting the use of olanzapine, fundamentally reshaped the prevention and management of CINV. These developments prompted a surge in randomized controlled trials, comparative effectiveness studies, and real-world evaluations designed to refine antiemetic regimens and optimize guideline-based care. In contrast, the observed decline in annual publications since 2020 may reflect a relative maturation of the field, with contemporary antiemetic combinations achieving high rates of symptom control and fewer transformative pharmacologic innovations emerging in recent years.

Importantly, a slowing of traditional CINV-focused research does not imply diminishing clinical relevance of nausea and vomiting as supportive care outcomes. Rather, it likely signals a shift in research emphasis as systemic cancer therapies evolve. Novel treatment modalities, including antibody–drug conjugates and immunotherapies, introduce distinct toxicity profiles that are not fully captured by conventional emetogenicity classifications. As such, future research may increasingly focus on characterizing and managing nausea and vomiting associated with these newer agents, as well as on patient-centered outcomes, real-world symptom burden, and persistent or refractory nausea despite guideline-concordant prophylaxis.

Several limitations merit consideration. First, our analysis relied on title and abstract screening and a single keyword-based search strategy, which may have missed relevant studies that examined CINV without explicitly using standardized terminology. Second, classification by emetogenicity was based on information available in abstracts, which may have led to misclassification in studies reporting heterogeneous regimens or incomplete treatment details. However, these limitations are mitigated by the large volume of included studies and the consistency of observed trends across decades. Moreover, the use of duplicate screening, of combining large language model–assisted review with independent human assessment, represents a pragmatic and scalable approach to literature surveillance, which may be increasingly valuable for meta-research in rapidly expanding fields.

In summary, the CINV literature has expanded substantially over time, driven largely by research on highly emetogenic chemotherapy and punctuated by periods of therapeutic innovation. As oncology continues to advance, maintaining a broad perspective on treatment-related nausea and vomiting—beyond traditional chemotherapy—will remain essential to optimizing supportive care and patient quality of life.

## Data Availability

No datasets were generated or analyzed during the current study.
